# Analysis of scientific production in spanish implantology

**DOI:** 10.4317/jced.53718

**Published:** 2017-05-01

**Authors:** Beatriz Tarazona, Antonio Vidal-Infer, Pablo Tarazona-Alvarez, Adolfo Alonso-Arroyo

**Affiliations:** 1 Department of Stomatology, School of Medicine and Dentistry, University of Valencia, Spain; 2 Department of the History of Science and Information Science, School of Medicine and Dentistry, University of Valencia, Spain

## Abstract

**Background:**

The aim of the study was to quantify the scientific productivity of researchers, organizations, and regions in Spain that publish articles on implantology in dental journals indexed in Journal Citation Reports.

**Material and Methods:**

A search was conducted among the core collection of Thomson Reuters’ Web of Science database, on the basis of its broad thematic and geographic coverage of health sciences. The search identified original articles – the main vehicle for the dissemination of research results. The search was conducted in July 2016, applying the truncated search term ‘implant*’ to locate original articles on implantology and its derivative forms. The search was conducted within the topic field (title, keywords and abstract) and two inclusion criteria were applied: documents denominated as articles were included; and articles categorized as Web of Science Medicine Dentistry and Oral Surgery. Finally only articles for which one of the participating organizations was located in Spain were selected.

**Results:**

The final search identified a total of 774 records. The period 1988 to 2015 saw an exponential growth in scientific production, especially during the last 10 years. Clinical Oral Implants Research and Medicina Oral Patologia Oral y Cirugia Bucal (Oral Medicine, Oral Pathology, and Oral Surgery) were the most productive journals. Collaborative networks among authors and among institutions increased and this increase was related to the improving quality of the publications.

**Conclusions:**

Bibliometric analysis revealed a significant growth in the quantity and quality of Spanish implantology literature. Most key bibliometric indicators demonstrated upward trends.

** Key words:**Bibliometric analysis, publication, keywords, implantology, implant.

## Introduction

Bibliometric indicators are statistical data extracted from the study of scientific publications. They offer a means of monitoring how the publication of new research findings contributes to the spread of knowledge, given that publishing marks every step in the scientific process (1).

As asserted by Moed *et al.* (2), bibliometric indicators are a worthwhile tool because they provide quantitative and concentrated information about both the production of knowledge and its impact. Despite their potential limitations, bibliometric indicators play an important role in evaluating research outcomes and the decision-making processes that determine scientific policy. There is sound evidence that bibliometric indicators are an important evaluation tool for both research groups and individual researchers (3).

In the field of dentistry, the last decade has seen technological progress grow hand in hand with the expansion of scientific publishing indicated, for example, by the fact that the number of articles on finite element analysis published within the field of dentistry is ten times greater than in other fields (4). This increase calls for ongoing bibliometric analysis in order to monitor scientific production. Several works have evaluated international scientific production in dentistry, including Kaur & Gupta (5) in India, and Gracio *et al.* (6) in Brasil. Other articles have focused on production at a national level, for example, the work by Bueno-Aguilera *et al.* (7) which provided an analysis of Spanish scientific production in dentistry from 1993 to 2012.

Nevertheless, studies of scientific production in implantology are scarce, and only one, by Tarazona *et al.* (8), evaluates global scientific production in implantology during the period 2009-2013.

To address the lack of bibliometric studies in this field, the present study provides an overview of scientific production in implantology in Spain. Bibliometric indicators were used to quantify the scientific productivity of researchers, organizations, and regions publishing articles on implantology in dental journals indexed in Journal Citation Reports.

## Material and Methods

-Search strategy

A search was conducted among the core collection of Thomson Reuters’ Web of Science database, selected on the basis of its broad thematic and geographic coverage of health sciences. The documents identified were all original articles – the main vehicle for the dissemination of research results.

The search took place in July 2016, applying the truncated search term “implant*” to locate original articles on implantology and its derivative forms. The search was conducted within the topic field (title, keywords and abstract) and two inclusion criteria were applied: documents denominated as articles; and articles categorized as Web of Science Medicine Dentistry and Oral Surgery. Articles about Orthodontics were excluded after a manual revision of title and abstract. Only articles in which one of the participating organizations was located in Spain were selected. The search identified a total of 774 articles that met all criteria.

All text files corresponding to the 774 articles were entered in a Microsoft Access database, using self-developed software Bibliometricos.

-Data Normalization

Records were manually refined and normalized to unify terms and to remove typographical, transcription and/or indexing errors; normalization was carried out in the fields ‘Author’, ‘Organization’, ‘Autonomous Region’ and ‘Province’.

The normalization process was complicated by the number of different entries for a single author. In these cases, the institutional affiliations of the authors were consulted to check whether different entries belonged to the same author. If this information was not available, an Internet search was carried out to eliminate any potential error.

Normalization of organizations followed the same procedure. Only macro-organizations (i.e., universities, research centers, etc.) were included, discarding micro-organizations, such as individual departments or research units. When the same organization signed the same work more than once, it was only counted once.

-Data analysis

Descriptive analysis of variables and a cross tabulation table were generated using Microsoft Access and Excel software. The evolution of scientific productivity by authors, organizations, regions, and journals was assessed. Analysis and visualization of large networks was performed using Pajek software (http://vlado.fmf.uni-lj.si/pub/networks/pajek/).

## Results

The final sample of articles consisted of 774 texts. The study period (1988 to 2015) saw an exponential growth in scientific production, especially during the last 10 years. From 1988 to 2007 there were less than 30 articles per year, then articles increased to 99 in 2014, reaching 147 in 2015.

-Scientific journals 

The articles were published in 48 different journals.  Table 1 shows the 10 most productive journals (with more than 15 articles); the first three contained the majority of publications, with more than 100 published works each. All journals published articles on dentistry and most of them were journals dealing with Surgery or Implants, with the exception of three on Periodontology and one on dental prosthetics. 26.7 % of them are ranked in the first quartile. The majority (7/10) were based in the USA, two in Denmark and one in Spain.

**Table 1:**
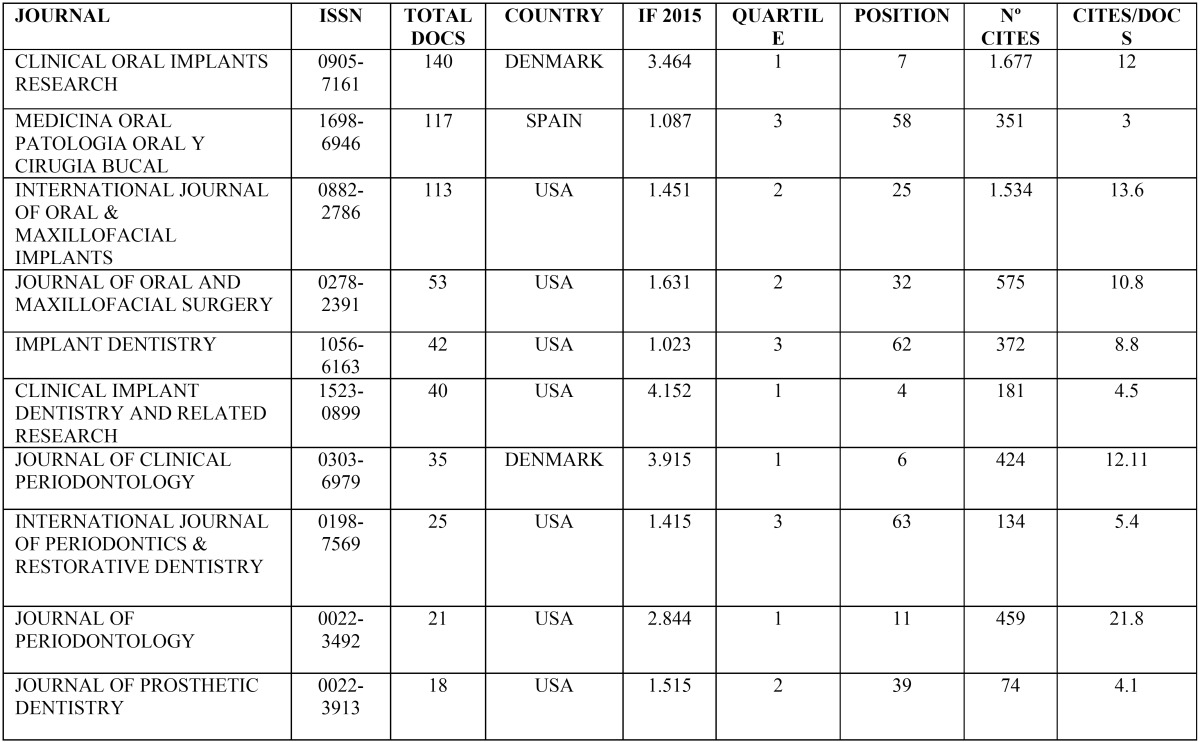
The most productive Journals (15 or more published documents).

Analyzing all 48 journals, 31% were ranked in the first quartile, 29% in the second quartile, 25% in the third quartile, 8% in the fourth quartile, and 6% were not ranked in journal citation reports (JCR) at the time when the search was performed. Most of the journal were based in the USA (24/48) and Europe (19/48), and only five in Asia.

The total number of Web of Science citations was 36,635. Analysis of citation data shows that the 10 most productive journals had more than 70 Web of Science citations. According to the present findings, Clinical Oral Implants Research (n=1,677) and the International Journal of Oral & Maxillofacial Implants (n=1,534) both had over 1,500 citations.

-Author production

A total of 4,078 signatures (mean: 5.27 signatures per article) belonging to 1,578 different authors were found in the 774 retrieved articles (mean: 2.04 authors per article). Following Bradford’s law of scattering to assess levels of productivity, distribution analysis found that when articles were grouped proportionally in three big areas, the proportion of authors varied from group to group. Each area had a value between 31% and 36% corresponding to the number of signatures, but the number of authors increased progressively. In this way, core productivity was driven by 65 authors with 10 or more articles, representing 4.1% of authors. In order to obtain the same number of works in the first area, a higher number of authors (279) were required (with a production of between three and nine works) representing 17.7% of authors. The last area corresponds to the authors with only 1 or 2 works, representing 1,234 authors (78.2% of the total).

 Table 2 shows the 29 most productive authors (with more than 15 published articles), highlighting the first two authors with more than 60 articles, Miguel Peñarrocha-Diago from the Universidad de Valencia with 82 articles, and Jose Luís Calvo-Guirado from the Universidad Católica San Antonio de Murcia (UCAM) with 74.

**Table 2:**
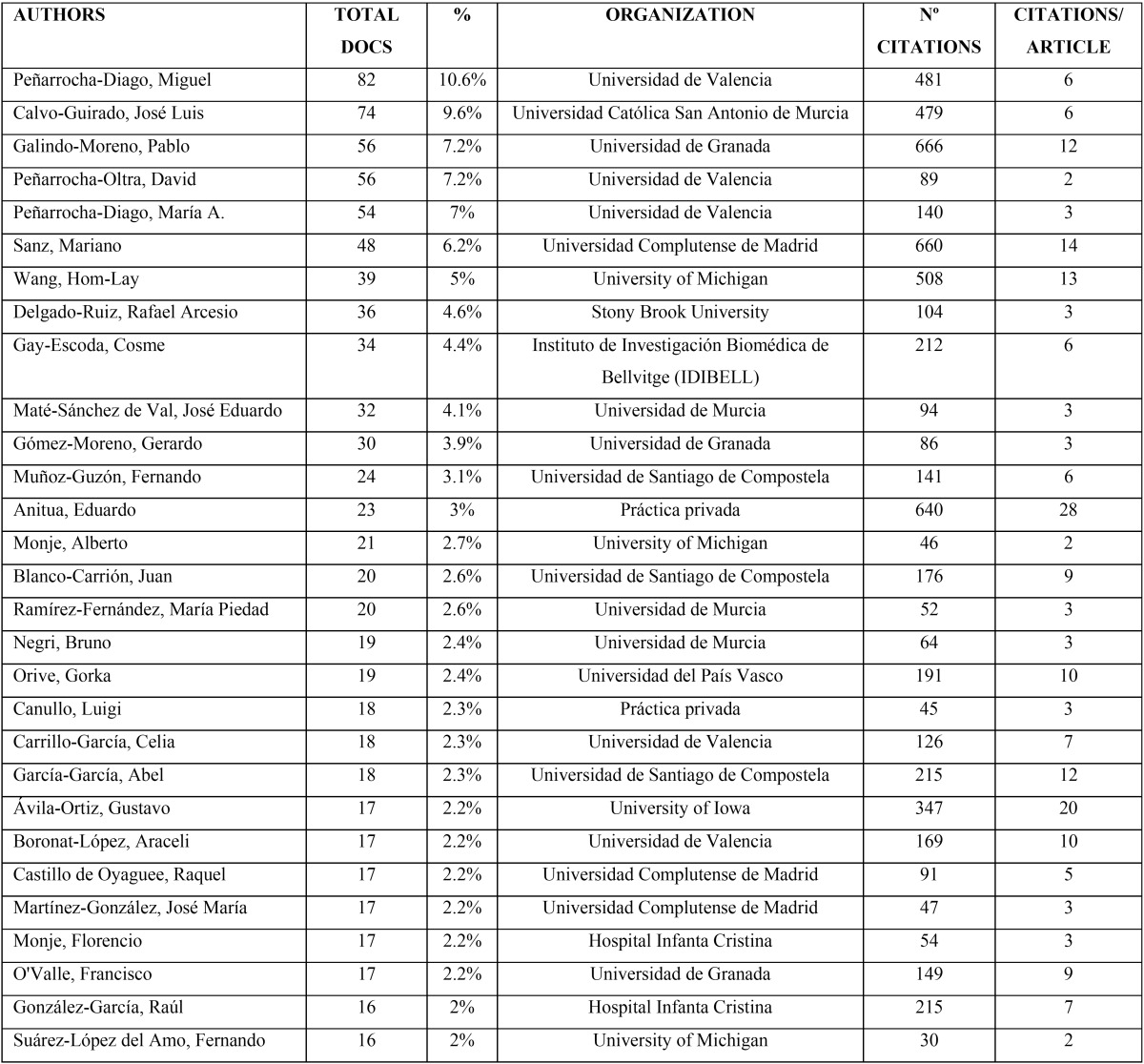
The most productive authors (more than 15 published documents).

The most productive authors had more than 45 citations making a total of 6,210 citations (17% of all citations). Analyzing the number of citations per article, the author with the most citations per article was not the most productive author; Eduardo Anitua has worked on 23 published articles with 640 citations (28 citations/article).

Over three quarters (78.3%) of the articles (n=606) received less than 10 citations. Nevertheless, analysis identified seven ‘hot’ papers with more than 100 citations ( Table 3). The most cited article was by Eduardo Anitua, who was also the author with the highest number of citations per document [358]. This author was also responsible for the article with the second highest number of citations per year (22.4 citations/year). Eduardo Anitua is a well-known specialist who has developed and applied the plasma-rich growth factor (PRGF) technique in various therapeutic situations; the article was published in 1999 and detailed and discussed the PRGF technique.

**Table 3:**
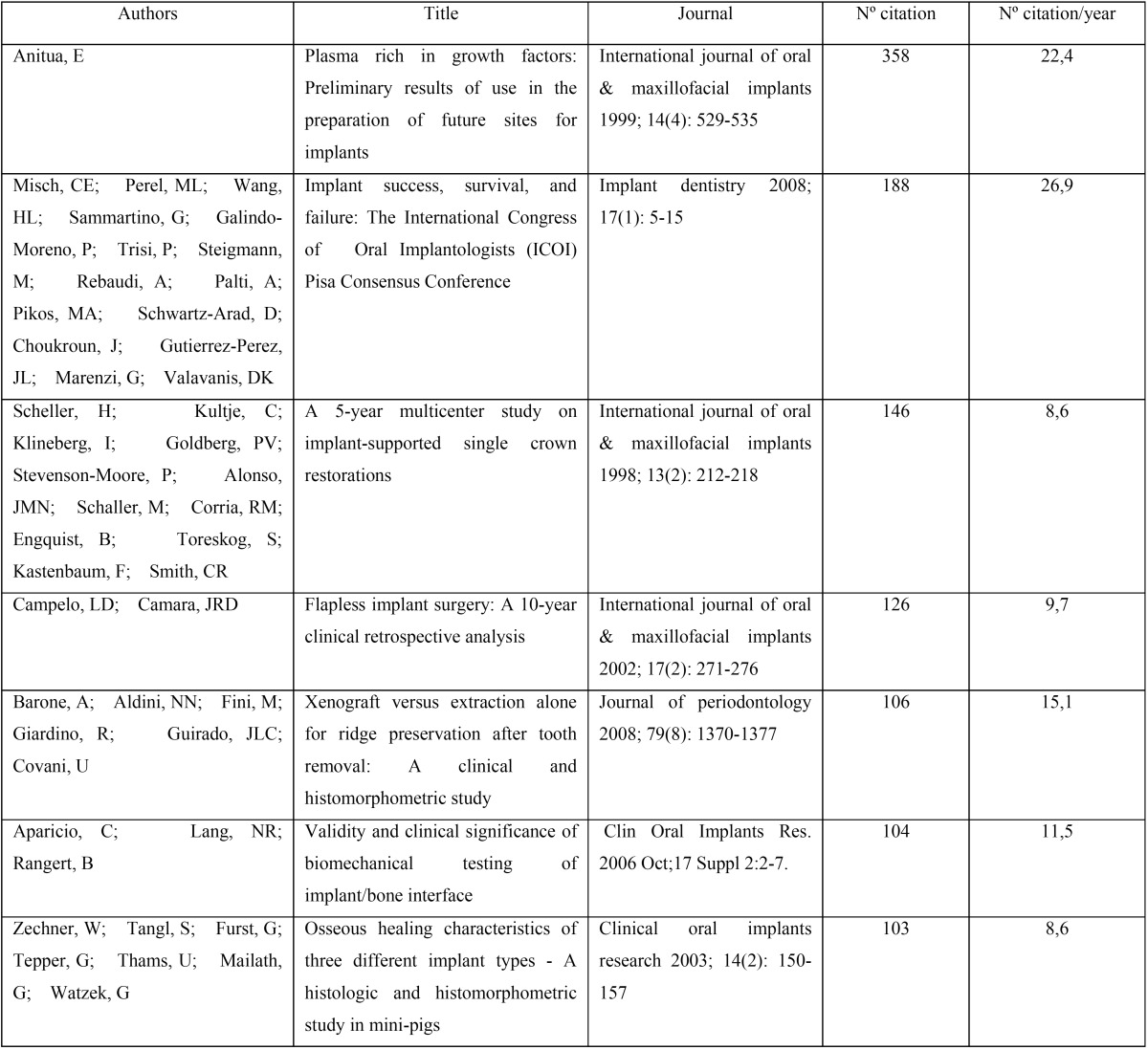
The seven ‘hot papers’ with more than 100 citations.

198 articles did not receive any citation.

**Figure 1:**
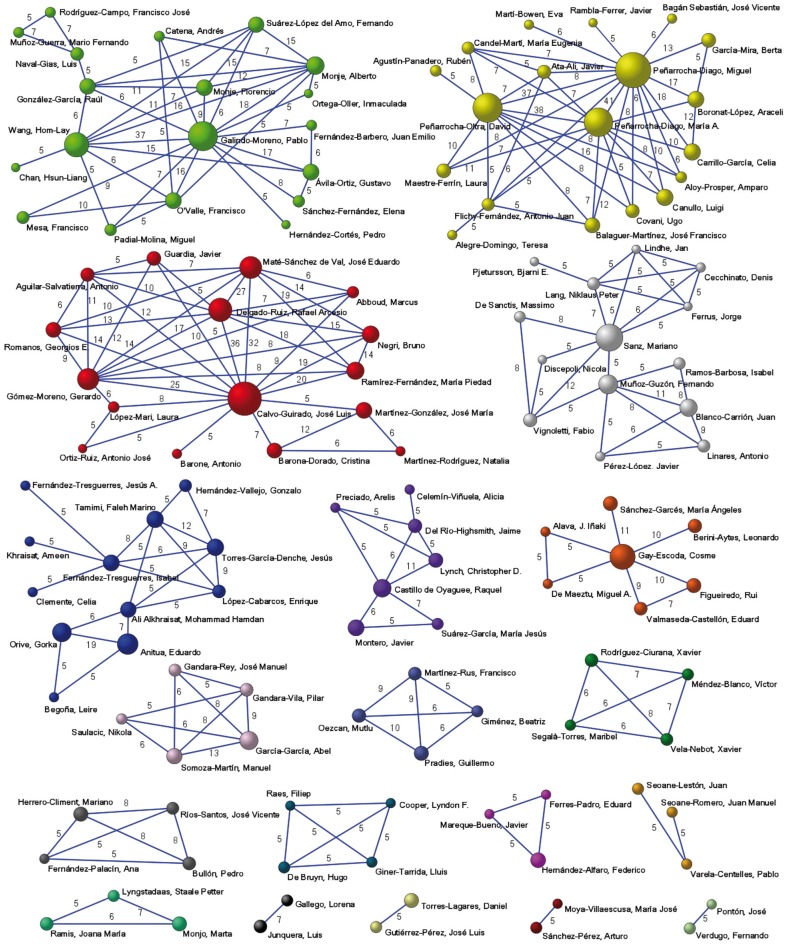
Authors’ collaborative networks (5 or more collaborations).

Figure 1 shows 15 research networks including 132 authors. The size of nodes (balls) located at the vertices is proportional to the number of articles published by each author. There are 4 main networks led by Miguel Peñarrocha-Diago from the University of Valencia (with 82 articles), José Luis Calvo-Guirado from the San Antonio University of Murcia (with 74 articles), Pablo Galindo-Moreno from the University of Granada (with 56 articles) and Mariano Sanz from the Complutense University of Madrid (with 48 articles). These nodes/vertices (authors) represent the lead authors of the 4 most significant research networks. Networks were mostly made up of authors from the same institutions, with the exception of collaborations, both national and international, with authors from other institutions.

In fact, 43 countries collaborated in Spanish production, with the USA collaborating in some 140 works. Figure 1 shows the four main networks with international collaborations.

The network led by Miguel Peñarrocha-Diago (University of Valencia) includes international authors such as Ugo Covani or Luigi Canullo from Italy. The same network is linked to two other big nodes, David Peñarrocha-Oltra (54) and María Peñarrocha-Diago (52) representing the fourth and fifth most productive authors.

The network led by José Luis Calvo-Guirado (San Antonio Catholic University of Murcia) also collaborates with an international author Marcus Abboud from the USA. The third network led by Pablo Galindo-Moreno (University of Granada) also includes international authors from the USA, Hom-Lay Wang and Hsun-Liang Chan. The network led by Mariano Sanz (Universidad Complutense de Madrid) includes the most international authors (7) from different countries including Italy, Iceland and China.

All 29 of the most productive authors were integrated in collaborative networks.

-Institutions

Analysis identified 360 institutions that participated in the articles, of which 136 were Spanish. The 14 most productive institutions, with more than 20 published works, are shown in  Table 4, and they are all Universities (except for the Hospital Quirón, Teknon), the most productive being the University of Granada (n=173), the Complutense University of Madrid (n=169), and the University of Valencia (n=160). Only two foreign institutions, the University of Michigan and the State University of New York, both from the USA, participated in Spanish articles. In relation to citations, the two most productive institutions received more than 1,000 citations, and the rest received more than 100 (with one exception). The Universidad de Oviedo shows the highest ratio of citations per article (n=9.65).

**Table 4:**
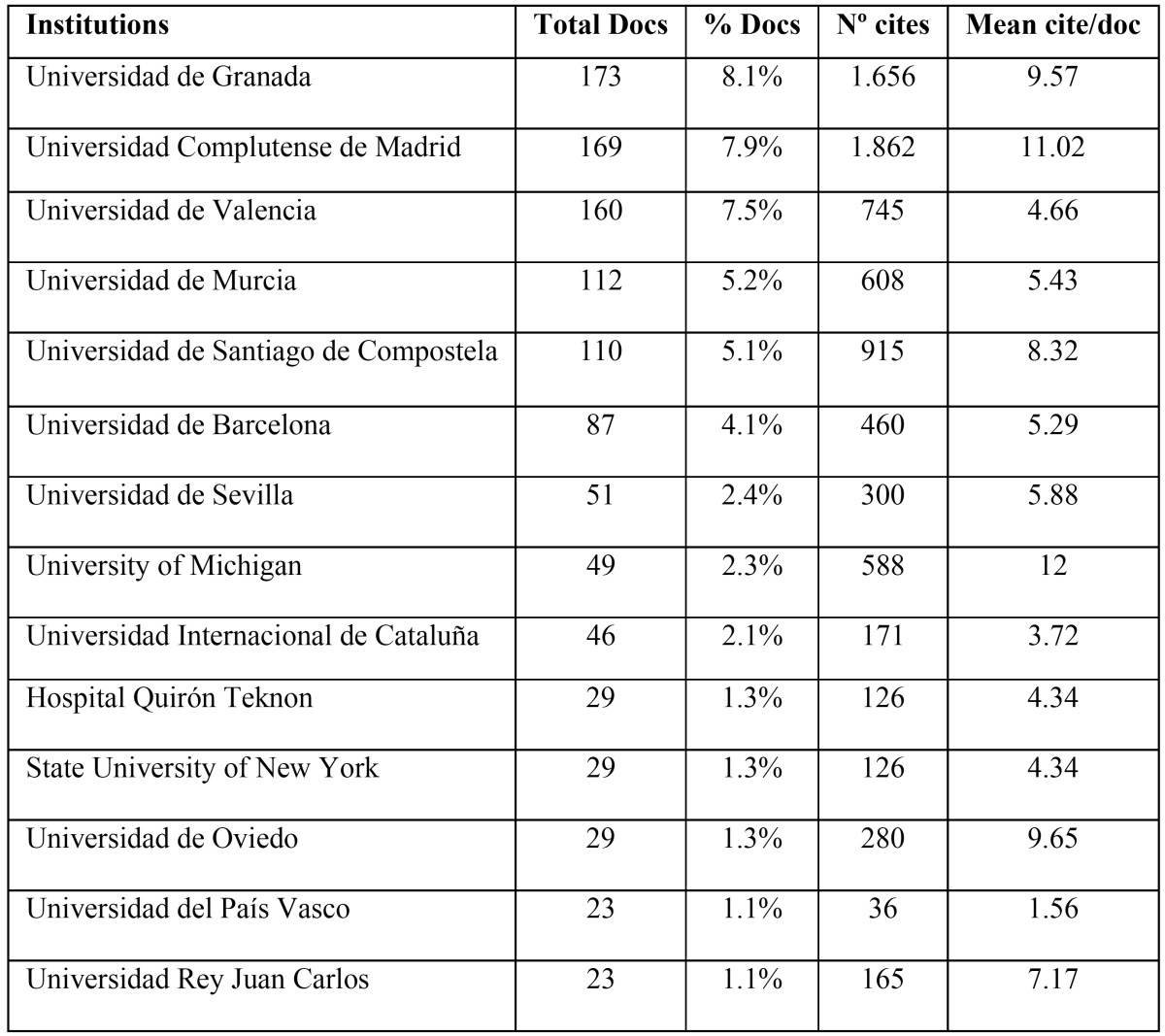
The most productive Institutions (20 or more published documents).

**Figure 2:**
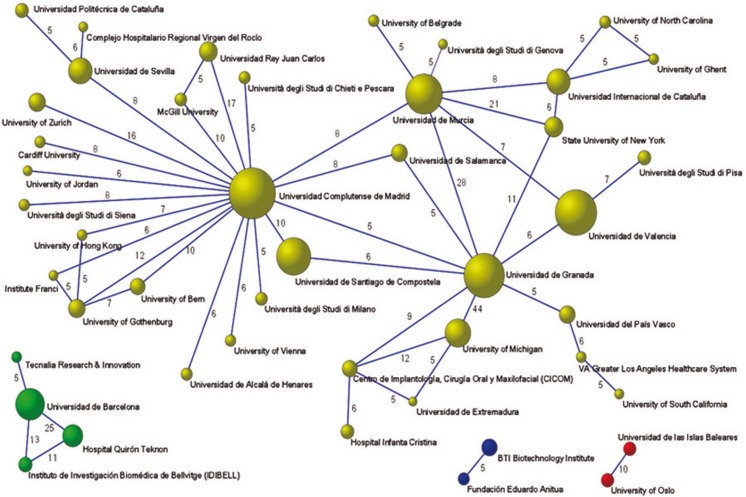
Institutional collaborative networks (5 or more collaborations).

Figure 2 illustrates networks of inter-organizational collaboration (defined by a threshold of at least five collaborations). The varying thickness of the links indicates the intensity of collaboration. The figure shows a large network of collaboration and three smaller networks that have produced at least five articles. The most productive institution, the Universidad de Granada has links with the Complutense University of Madrid and the University of Valencia, although the latter are not directly linked.

Foreign institutions are also linked into Spanish institutional networks, for example, the University of Granada and the University of Michigan, who have collaborated on 44 works, or the University of the Balearic Islands and Oslo University, who have collaborated on ten articles.

-Geographic production

Over the period analyzed (1988-2015), 43 different producer countries were identified, most of them European (n=26), eight American, six Asian, two African and one in Oceania. Analyzing production per country, the most collaborative countries with Spain were the USA with 189 articles (8.8%), Italy with 100 articles (4.7%), Germany with 72 articles (3.4%), and Sweden with 58 articles (2.7%).

**Figure 3:**
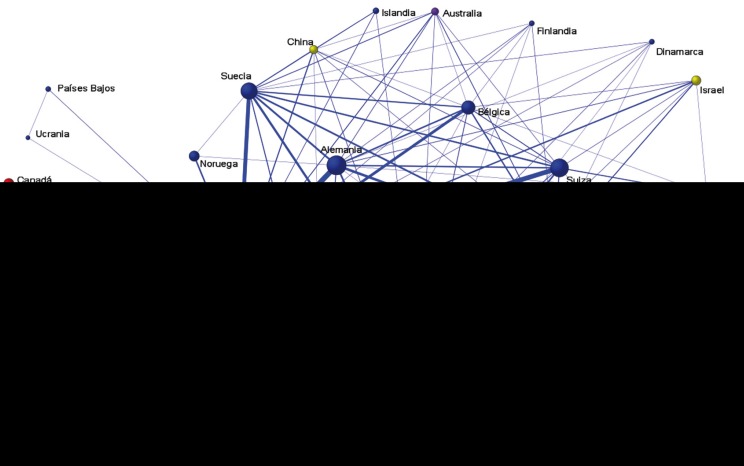
International collaborative networks (5 or more collaborations).

Figure 3 shows that Spain represents the biggest size node, followed by the USA and Italy. Some countries only collaborated with Spain or USA, such as France, Lithuania, Serbia, or Brazil. Nevertheless, other countries such as Germany, Belgium or Switzerland, collaborate with many other countries.

All the Spanish provinces, with the exception of the region of Cantabria, produced articles.

Within Spain, the Province of Madrid (n=286), Region of Andalucia (n=242) and Catalonia (n=194) were the most productive Autonomous Regions with more than 10% of published work each. Then, Comunidad Valenciana (n=194), Galicia (n=119) and Region of Murcia (n=116).

Thirty of the Spanish provinces produced articles. Analysis of production per province shows that Madrid (n=286) and Barcelona (n=236) were the most productive with more than 10% of published work each. Then, Granada (n=180), Valencia (n=173) and Murcia (n=116) with a percentage of published work between 10%-5% each one.

## Discussion

Spanish scientific production in implantology has grown progressively over the period analyzed (1988-2015), especially during the last 10 years. The causes of this phenomenon reside not only in the growth and development of Spanish implantology, as a science with an increasing number of potential participants in the research process, but also in other factors such as the introduction of new technologies, as in other dentistry fields (7).

The study included articles published in 48 journals, including one Spanish journal. Other journals were mainly from the USA, except for two Danish journals, a fact that points to the quality and internationalization of Spanish implantology. The number of articles cited in other papers (n= 36,635) is another indication of the quality of Spanish scientific production.

The journals publishing the articles were mainly publications on implantology, although some articles also appear in journals focusing on periodontology and prosthetics.

As could be expected, the most productive authors are internationally renowned professionals. Some of them work as private clinicians, but almost all of them belong to universities. The most productive author is Miguel Peñarrocha-Diago, from the University of Valencia, with 82 articles (10.6% of the total).

The mean number of signing authors per article was 2.04, a slightly lower figure than in other fields (8,9); this maybe due to the early start-date of the period analyzed (1988), since the number of signatures per article has grown annually. This progressive increase might be due to a growing need for larger numbers of participants in research initiatives in order to conduct innovative studies of quality, a tendency that has been observed in other specialized fields (7).

A significant finding of the present analysis is that the most productive authors (with 15 works or more) represent 21.4% of the signatures and 33.8% are transitory authors with only one signed work. In many cases, transitory authors publish circumstantially or do not belong to any scientific institution as they work as private clinicians, a common occurrence in other medical specialties (10). As a matter of fact, the most cited article (358 citations) was signed by Eduardo Anitua as a single author, who is not affiliated to any University.

As could be expected, the most productive authors lead collaborative networks, based on collaborations among authors belonging to same institution or region. Current publishing difficulties require increasing levels of collaboration, a phenomenon that also affects other dentistry specialties (11).

The most productive institutions (with 20 works or more) were mostly universities with the exception of a number of hospitals. The most productive was the University of Granada (n=173), the Complutense University of Madrid (n=169) and the University of Valencia (n=160), as in other specialties (7).

Other data confirm the internationalization of Spanish implantology. The four main collaboration networks have links to international universities. Moreover, the list of collaborations includes the University of Michigan and the State University of New York.

The countries engaged in the highest numbers of collaborations with Spain were the USA (8.8%), Italy (4.7%), Germany (3.4%) and Sweden (2.7%), a finding that concurs with other studies (7). This fact is confirmed by the configuration of the collaboration networks analyzed.

Regarding total production by region, the study found that the province of Madrid, and the regions of Andalucía, Catalonia and Valencia were the most productive autonomous regions, as seen in other medical specialties (10). Furthermore, their capitals were the most productive cities, with the exception of Granada, which was the most productive city in Andalucía. This geographical distribution coincides with data about the most productive institutions and authors.

In conclusion, the present bibliometric analysis of Spanish scientific production in the field of implantology shows that production has increased exponentially in terms of author numbers, articles, and quality. The data obtained also demonstrate the internationalization and increased levels of collaboration with international researchers.
